# Supplementary material for: Structure-guided mutagenesis of OSCAs reveals differential activation to mechanical stimuli
Source: bioRxiv. 2024 Jan 13:2023.10.03.560740. Originally published 2023 Oct 3. Preprint. [Version 2] doi: 10.1101/2023.10.03.560740 (PMC10592937; doi:10.1101/2023.10.03.560740)
Supplement: Supplementary file 1 [file media-1.pdf]

Supplemental figures

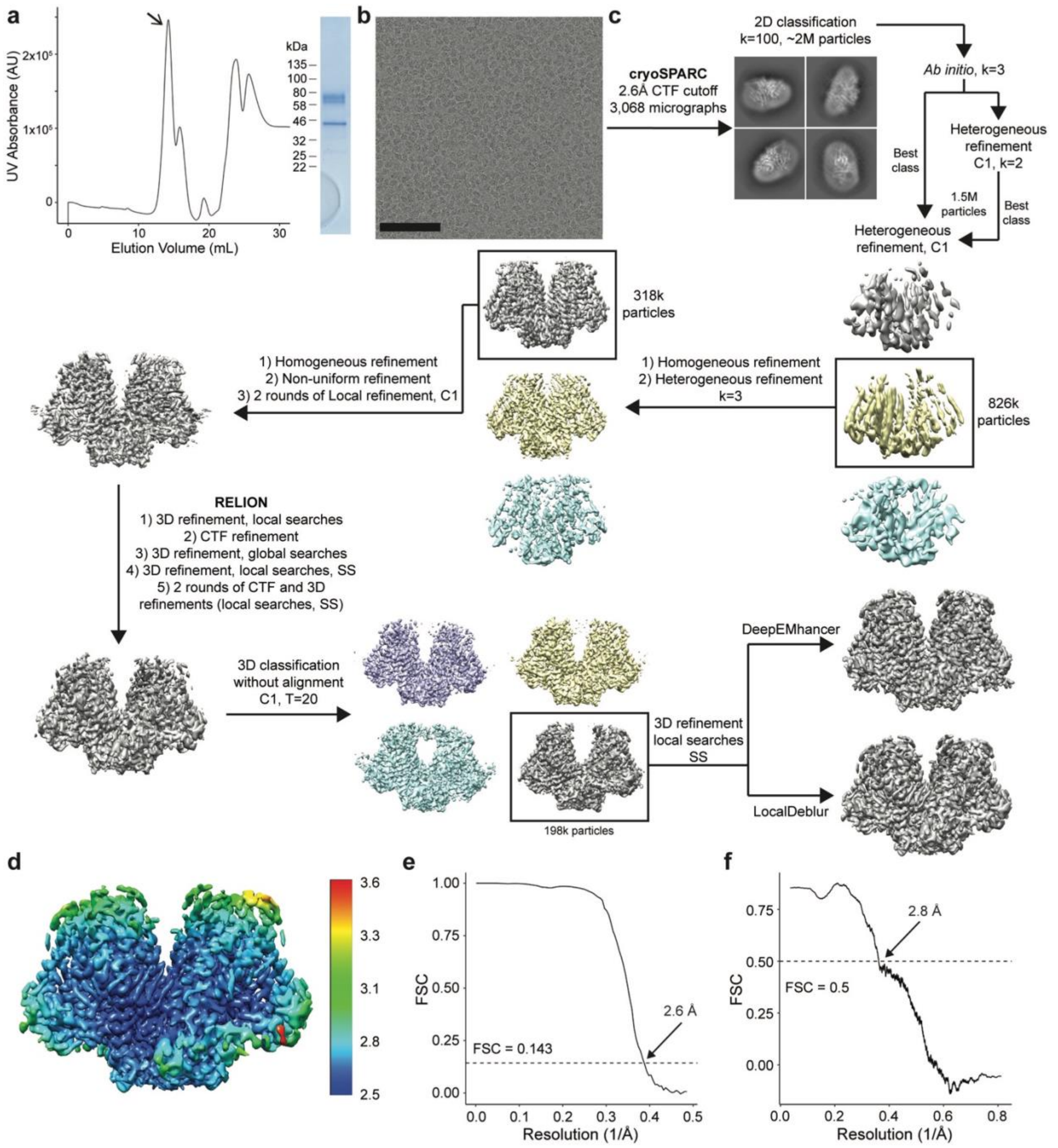

Supplementary Figure 1: Purification and cryo-EM data processing of OSCA3.1. **a,** SEC trace (left) from OSCA3.1 reconstitution in nanodiscs. Arrow points to peak corresponding to fractions pooled and ran on SDS-PAGE (right). Molecular weight of OSCA3.1 and MSP2N2 is approximately 82kDa and 43kDa, respectively. **b,** Representative cryo-EM micrograph. Black bar is 100nm. **c,** Cryo-EM processing workflow. C2 symmetry was imposed during refinements unless otherwise specified. 3D refinements in RELION were performed using the SIDESPLITTER algorithm where

indicated (SS). **d,** LocalDeblur map colored by local resolution calculated in RELION. **e,** FSC plot calculated in RELION. **f,** LocalDeblur map to model FSC plot.

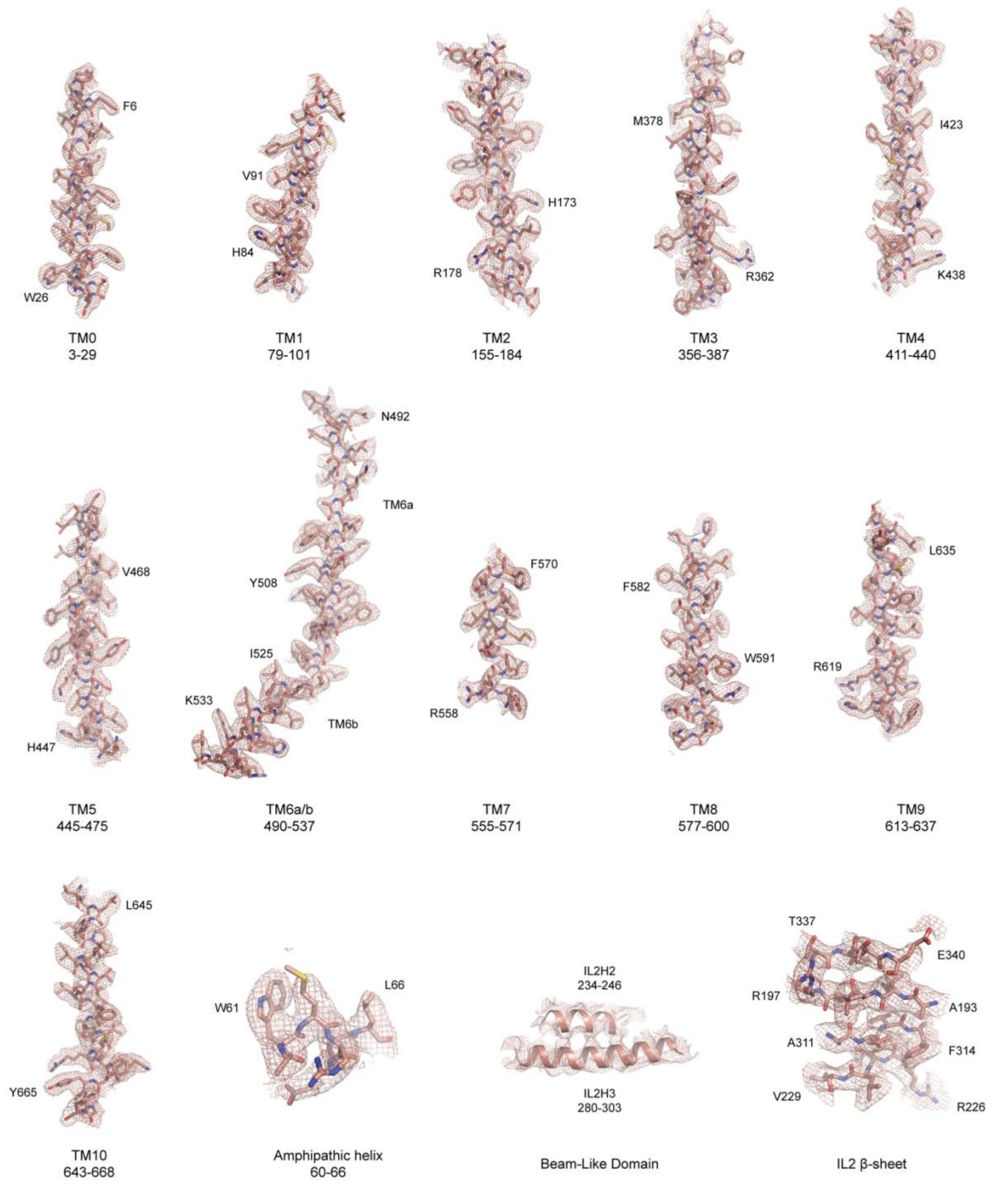

Supplementary Figure 2: Fit of OSCA3.1 model to LocalDeblur map.
Fit of OSCA3.1 model to selected regions of the LocalDeblur map. Map was contoured at a threshold of 2 (for the BLD) or 4 σ.

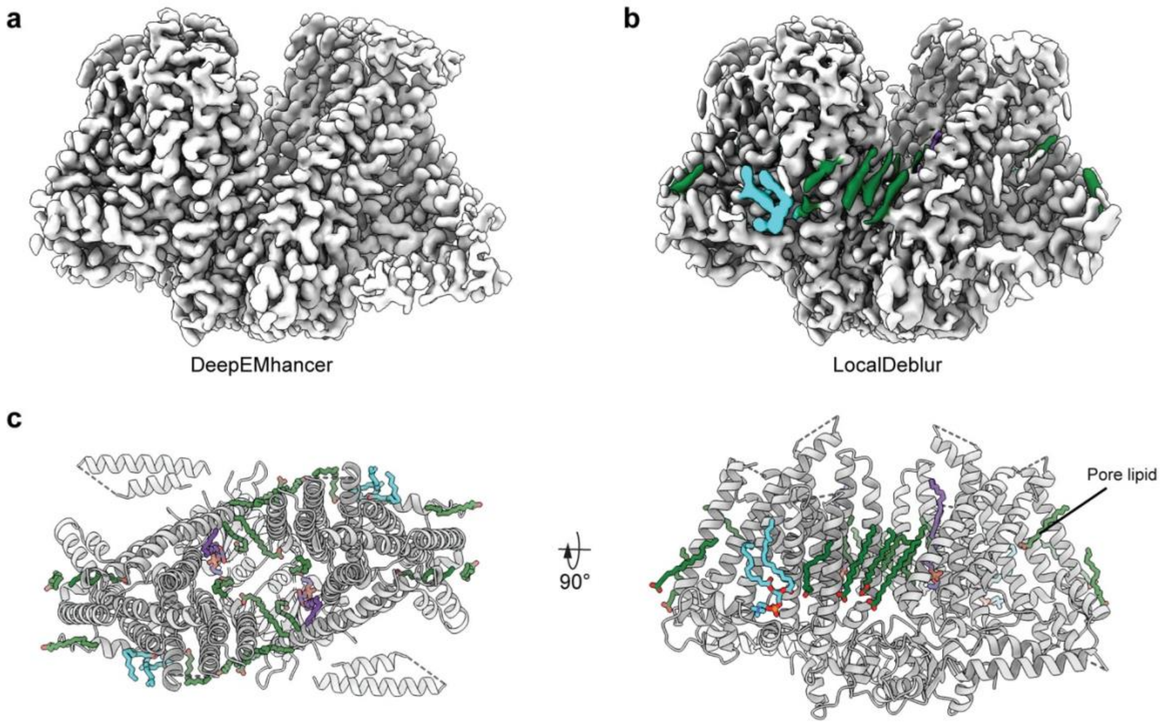

Supplementary Figure 3: Comparison of DeepEMhancer and LocalDeblur postprocessed maps.
a, DeepEMhancer map. **b,** LocalDeblur map with lipid-like densities colored. **c,** Top (left) and front (right) view of OSCA3.1 model with modelled lipids. Lipids were tentatively modelled as palmitic acid (green), phosphatidylcholine (cyan), and lyso-phosphatidylethanolamine (purple).

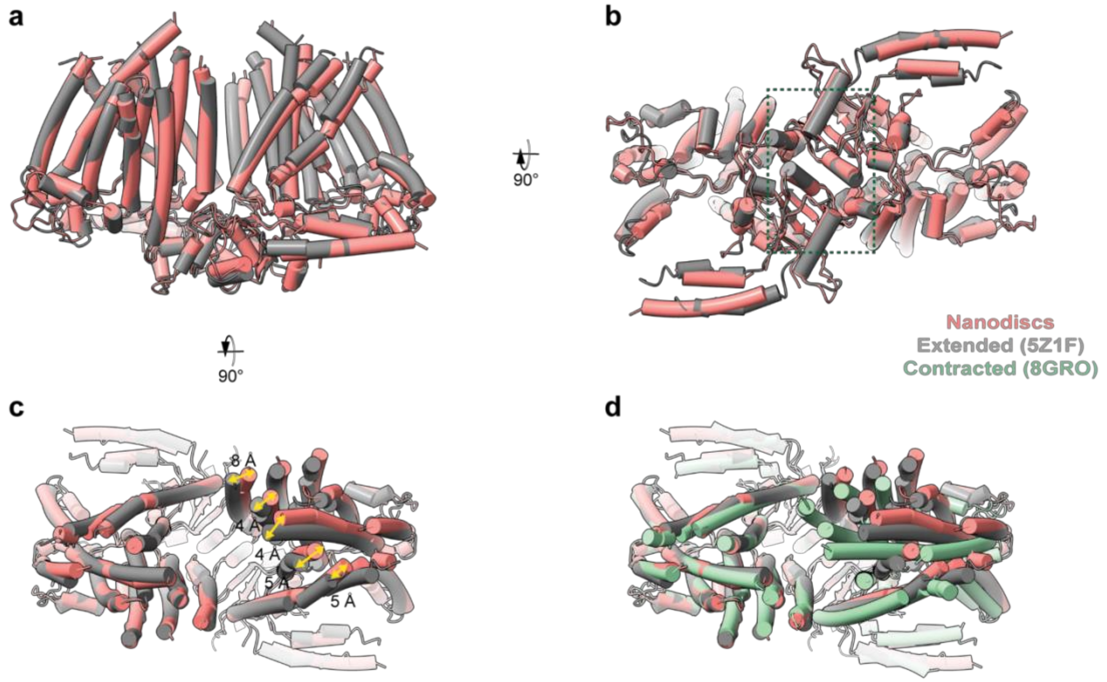

Supplementary Figure 4: Comparison of OSCA3.1 in nanodiscs and detergent states.
a, Front view of model superposition of OSCA3.1 in nanodiscs and extended state in digitonin (PDB: 5Z1F). Alignment done on left protomer. **b,** Intracellular view. Dimerization domain is enclosed in a green square. **c,** Extracellular view illustrating shifts of the nanodisc sample towards the outer edges, and increasing the distance between the two protomers in the inter-subunit cleft. **d,** Extracellular view of the OSCA3.1 in nanodiscs, extended state in digitonin, and contracted state in LMNG (PDB: 8GRO).

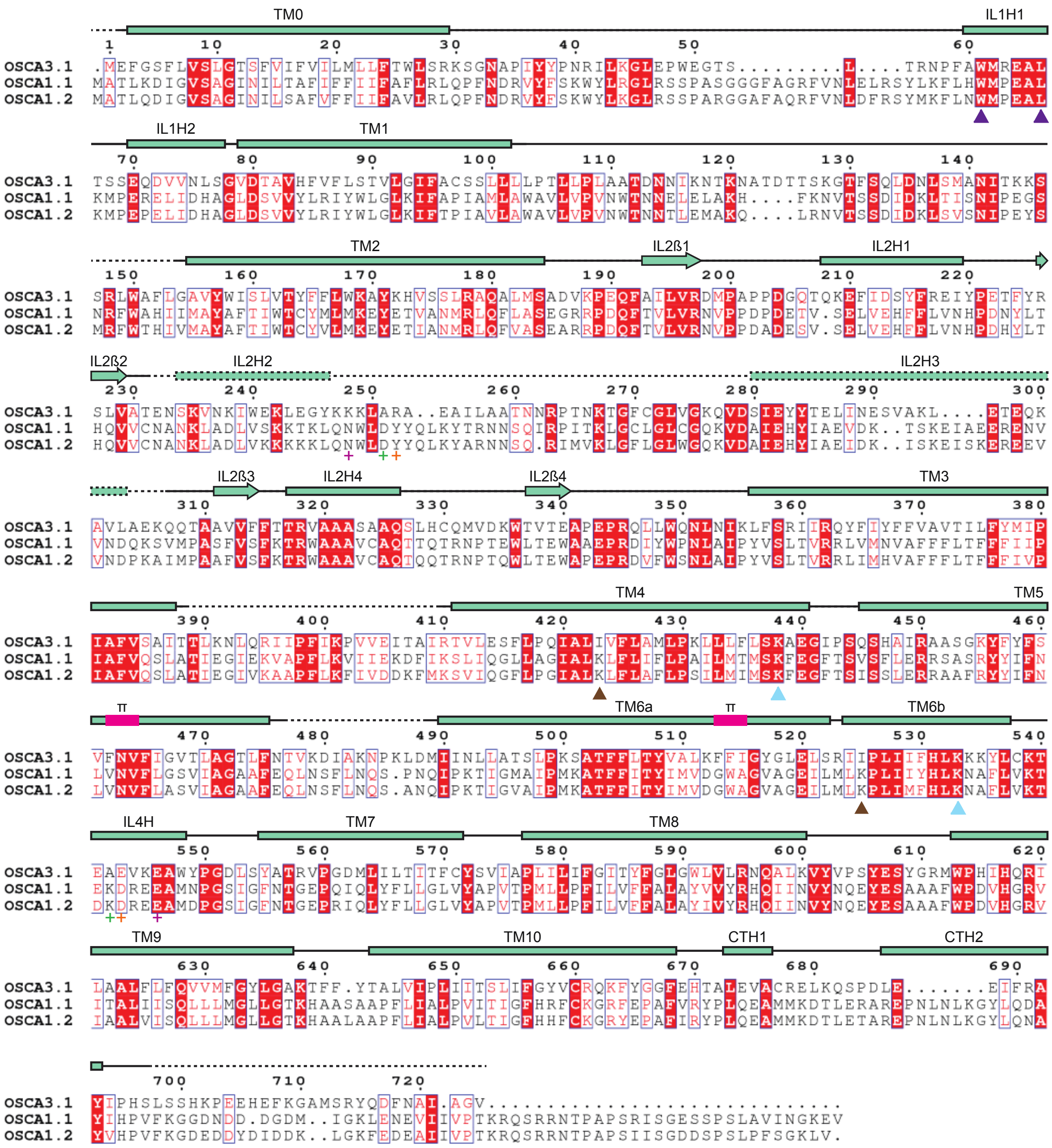

Supplementary Figure 5: Protein sequence alignment of OSCA3.1.
Amino acid sequence alignment of *Arabidopsis thaliana* OSCA3.1, OSCA1.1 and OSCA1.2. Secondary structure of OSCA3.1 represented on top, where rectangles represent α helices and arrows β strands. Dashed lines correspond to regions not modelled. π-helical turns labeled in magenta. IL2H2 and IL2H3 were modelled as poly-Ala and the registry was assigned tentatively. Purple arrowheads point to residues in the amphipathic helix that were mutated in OSCA1.2 for electrophysiology experiments. Predicted lipid-interacting residues denoted by light blue (conserved between OSCA1.2 and OSCA3.1) or brown (not conserved) arrowheads. Evolutionarily coupled residues

marked by plus sign (+), with each pair having the same color. TM: Transmembrane; IL: Intracellular Loop; CT: C-terminal; H: Helix; ß: ß-strand.

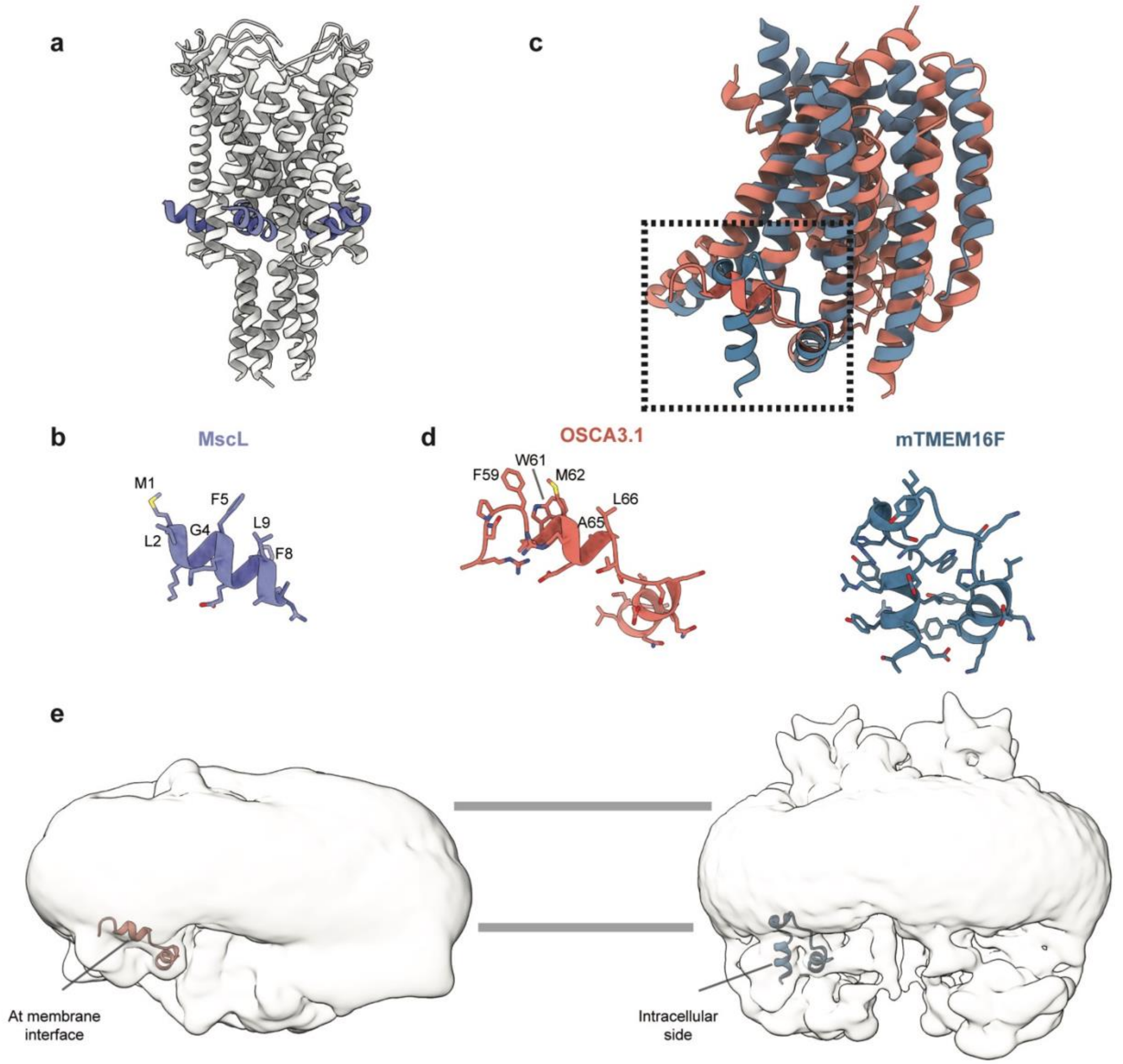

Supplementary Figure 6: Comparison of the amphipathic helix of OSCA3.1 with corresponding helices in TMEM16 structures.
a, Structure of MscL (PDB:2OAR). The amphipathic helix is highlighted in purple. **b,** Amphipathic helix of MscL with lipid-facing residues labeled. **c,** Superposition of transmembrane region of one protomer of OSCA3.1 (TM0 was excluded for simplicity) and homologous mouse (m) TMEM16F (PDB:6QP6). **d,** Inset from **c**: zoom-in view of amphipathic helix of OSCA3.1 (left) and homologous intracellular helix of mTMEM16F (right). Lipid-facing residues of OSCA3.1 are labeled. **c,** EM density of unsharpened density of OSCA3.1 (left) and mTMEM16F (right, EMDB: 4611) showing the position of respective helices shown in **c** and **d**. A gaussian-filter of 2 σ was applied to the densities. Horizontal gray lines denote the approximate position of the membrane bilayer.

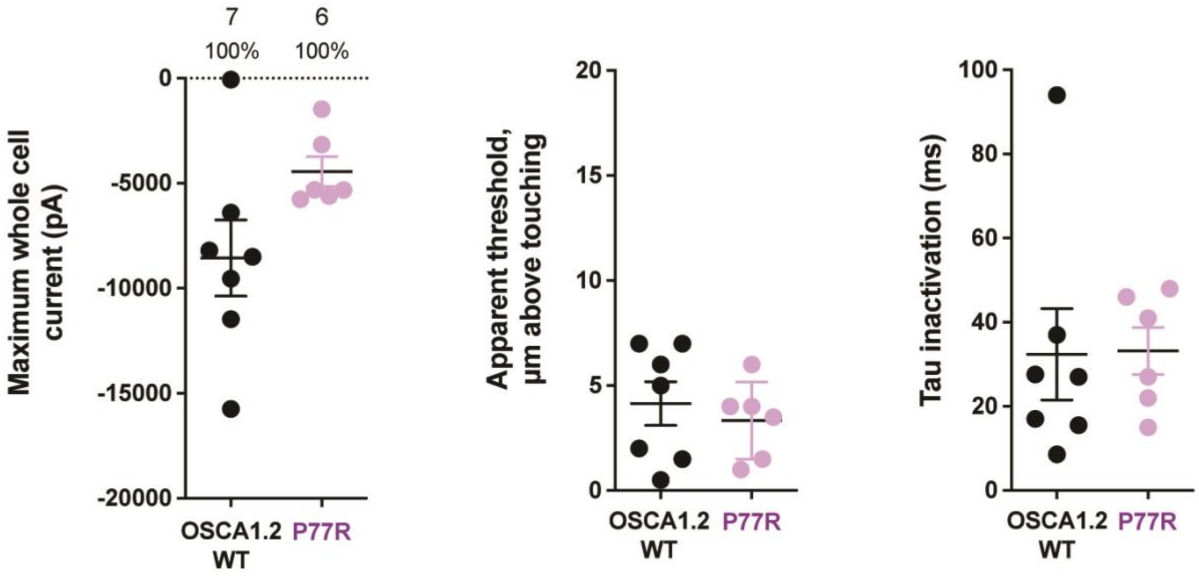

Supplementary Figure 7: Poke-induced response of OSCA1.2$_{P77R}$.
Introduction of arginine from OSCA3.1 into the OSCA1.2 background did not alter the poke-induced response. Left panel: Maximum poke-induced whole-cell currents observed for cells exposed to displacements up to 7.2 ± 1.4 µm (N=7; mean ± S.E.M.) and 6.5 ± 0.6 µm (N=6) above touching for WT and OSCA1.2$_{P77R}$, respectively. Center panel: the apparent threshold in µm above touching for the cells in this cohort. Right panel: Time constant of inactivation of currents elicited in the cells in this cohort.

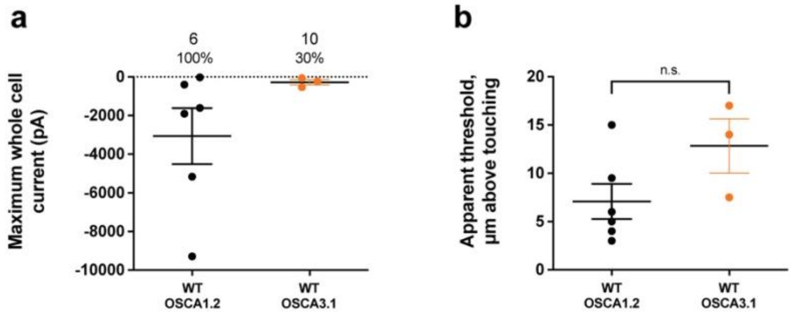

Supplementary Figure 8: OSCA3.1 may be poke-sensitive at high thresholds. Poke-induced whole-cell current responses are observed in HEK-P1KO cells expressing OSCA3.1. **a,** Maximum poke-induced currents observed in whole-cell mode for cells exposed to displacements up to 10.4 ± 1.2 µm (N=6; mean ± S.E.M.) and 13.8 ± 0.9 µm (N=3) above touching for OSCA1.2$_{WT}$ and OSCA3.1$_{WT}$, respectively. Although 6 of 6 OSCA1.2 expressing cells responded to poking, only 3 of 10 showed a response before the disruption of the recording. **b,** The apparent threshold in µm above touching the cell for this cohort.

Supplementary Table 1. Data collection, processing, model refinement and validation.

	OSCA3.1
**Data collection and processing**	
Magnification	29000
Voltage (kV)	300
Electron exposure (e–/Å^2)	50
Defocus range (μm)	-0.4 to -1.5
Pixel size (Å)	1.03
Initial particle images (no.)	1,913,316
Symmetry imposed	C2
Final particle images (no.)	197,944
Map resolution (Å)	2.6
FSC threshold	0.143
Map sharpening *B* factor (Å^2)	-64
**Model**	
Composition	
Peptide chains	2
Protein residues	1148
Ligands	24
R.m.s. deviations	
Bond lengths (Å)	0.023
Bond angles (°)	1.677
Validation	
MolProbity score	0.74
Clashscore	0.74
EMRinger score	4.12
Poor rotamers (%)	0.00
Ramachandran plot	
Favored (%)	98.93
Allowed (%)	1.07
Disallowed (%)	0.00
**Deposition ID**	
EMDB	41911
PDB	8U53
