# Supplementary material for: Structure-guided mutagenesis of OSCAs reveals differential activation to mechanical stimuli
Source: bioRxiv. 2023 Oct 3:2023.10.03.560740. Preprint. [Version 1] doi: 10.1101/2023.10.03.560740 (PMC10592937; doi:10.1101/2023.10.03.560740)
Supplement: Supplementary file 1 [file NIHPP2023.10.03.560740v1-supplement-1.pdf]

bioRxiv preprint doi: https://doi.org/10.1101/2023.10.03.560740; this version posted October 3, 2023. The copyright holder for this preprint (which was not certified by peer review) is the author/funder, who has granted bioRxiv a license to display the preprint in perpetuity. It is made available under aCC-BY 4.0 International license.

Supplemental figures

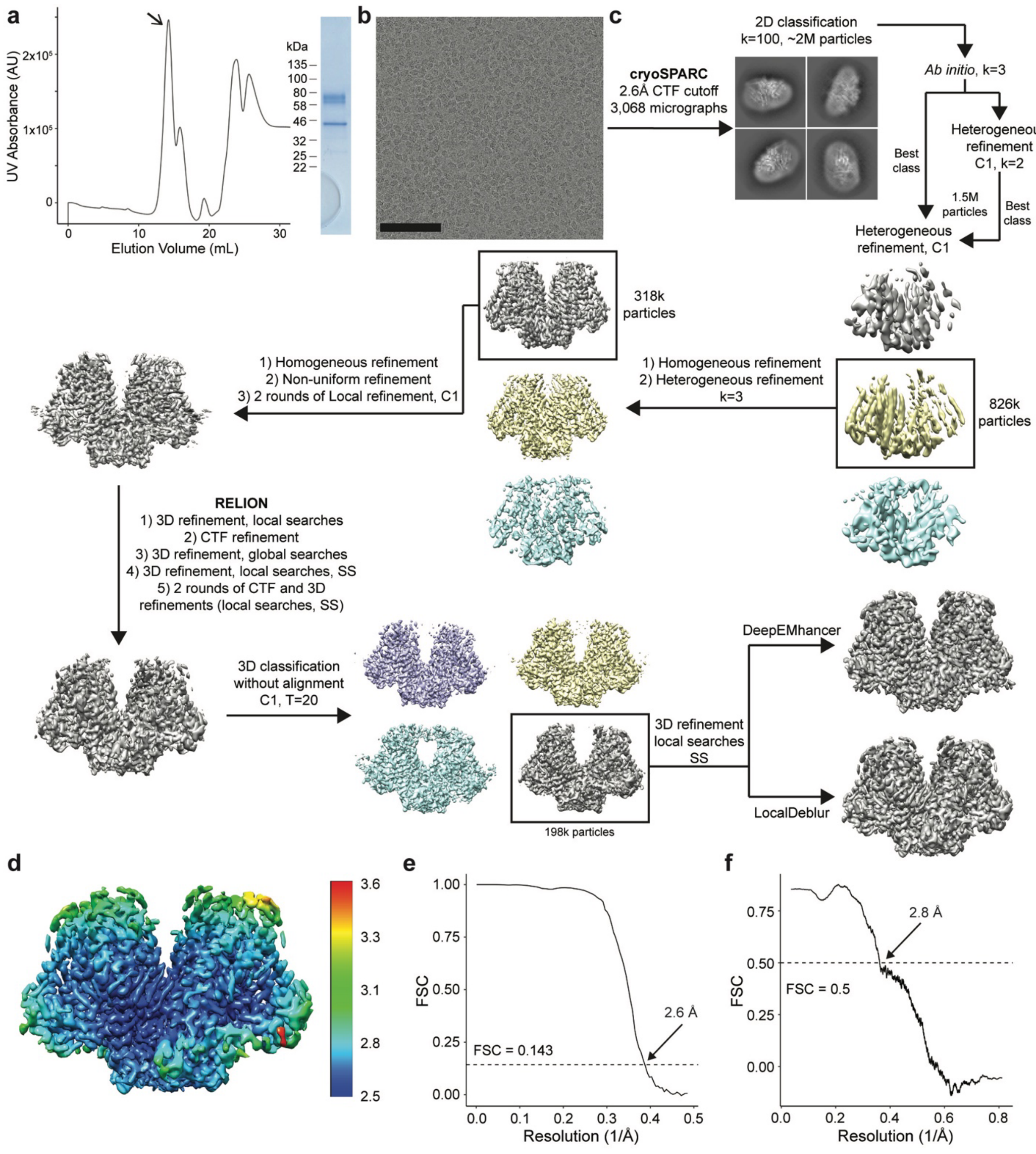

Supplementary Figure 1: Purification and cryo-EM data processing of OSCA3.1. **a,** SEC trace (left) from OSCA3.1 reconstitution in nanodiscs. Arrow points to peak corresponding to fractions pooled and ran on SDS-PAGE (right). Molecular weight of OSCA3.1 and MSP2N2 is approximately 82kDa and 43kDa, respectively. **b,** Representative cryo-EM micrograph. Black bar is 100nm. **c,** Cryo-EM processing workflow. C2 symmetry was imposed during refinements unless otherwise specified. 3D refinements in RELION were performed using the SIDESPLITTER algorithm where

bioRxiv preprint doi: https://doi.org/10.1101/2023.10.03.560740; this version posted October 3, 2023. The copyright holder for this preprint (which was not certified by peer review) is the author/funder, who has granted bioRxiv a license to display the preprint in perpetuity. It is made available under aCC-BY 4.0 International license.

indicated (SS). **d,** LocalDeblur map colored by local resolution calculated in RELION. **e,** FSC plot calculated in RELION. **f,** LocalDeblur map to model FSC plot.

bioRxiv preprint doi: https://doi.org/10.1101/2023.10.03.560740; this version posted October 3, 2023. The copyright holder for this preprint (which was not certified by peer review) is the author/funder, who has granted bioRxiv a license to display the preprint in perpetuity. It is made available under aCC-BY 4.0 International license.

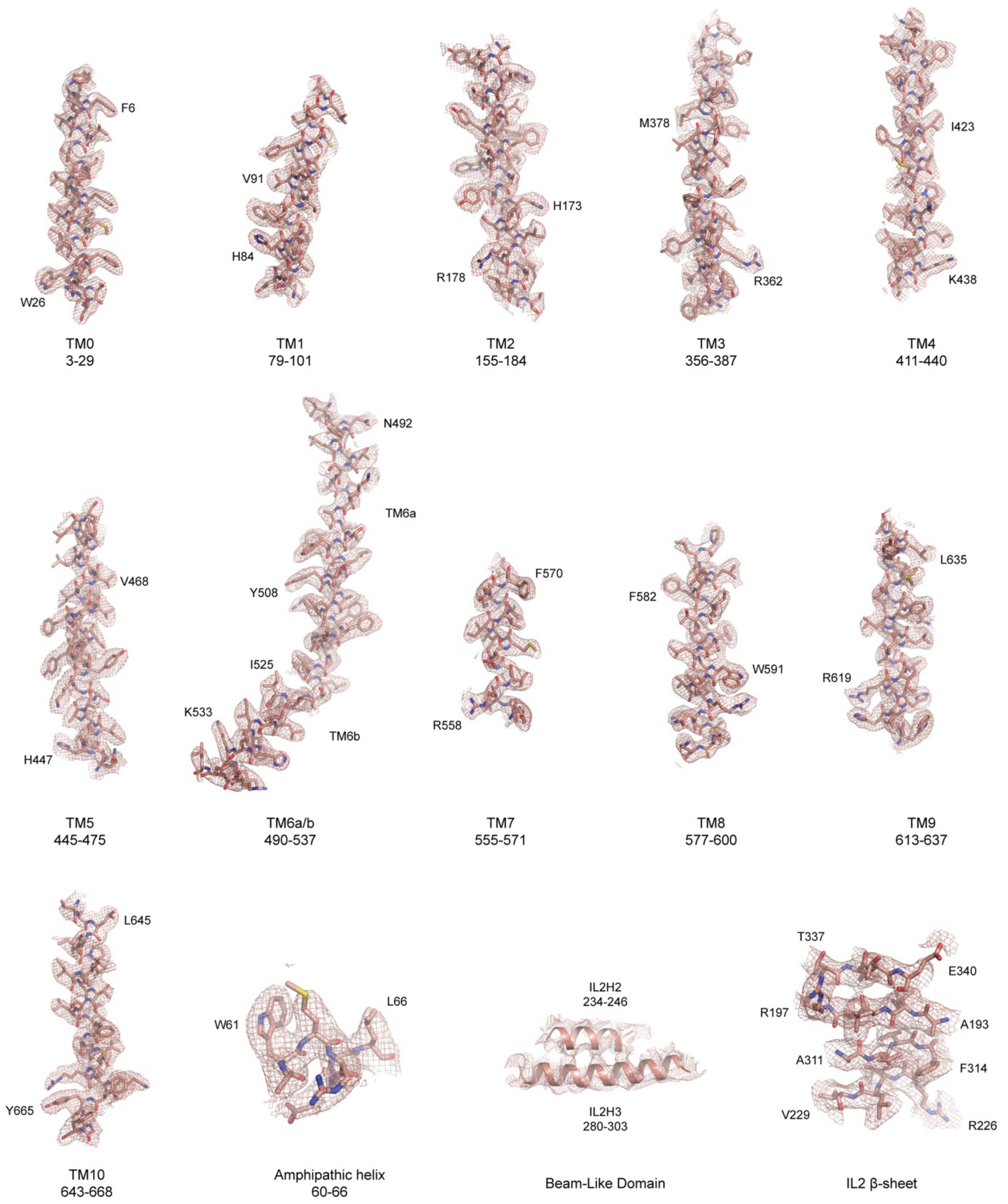

Supplementary Figure 2: Fit of OSCA3.1 model to LocalDeblur map.
Fit of OSCA3.1 model to selected regions of the LocalDeblur map. Map was contoured at a threshold of 2 (for the BLD) or 4 σ.

bioRxiv preprint doi: https://doi.org/10.1101/2023.10.03.560740; this version posted October 3, 2023. The copyright holder for this preprint (which was not certified by peer review) is the author/funder, who has granted bioRxiv a license to display the preprint in perpetuity. It is made available under aCC-BY 4.0 International license.

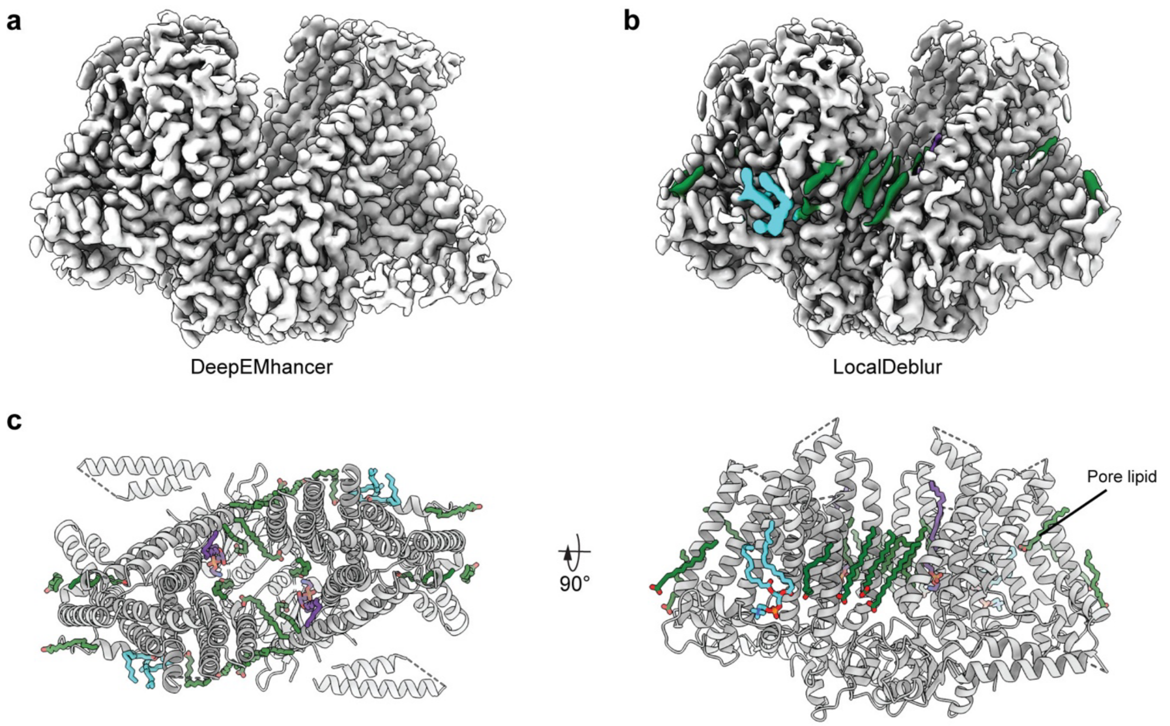

Supplementary Figure 3: Comparison of DeepEMhancer and LocalDeblur postprocessed maps.

a, DeepEMhancer map. **b,** LocalDeblur map with lipid-like densities colored. **c,** Top (left) and front (right) view of OSCA3.1 model with modelled lipids. Lipids were tentatively modelled as palmitic acid (green), phosphatidylcholine (cyan), and lyso-phosphatidylcholine (purple).

bioRxiv preprint doi: https://doi.org/10.1101/2023.10.03.560740; this version posted October 3, 2023. The copyright holder for this preprint (which was not certified by peer review) is the author/funder, who has granted bioRxiv a license to display the preprint in perpetuity. It is made available under aCC-BY 4.0 International license.

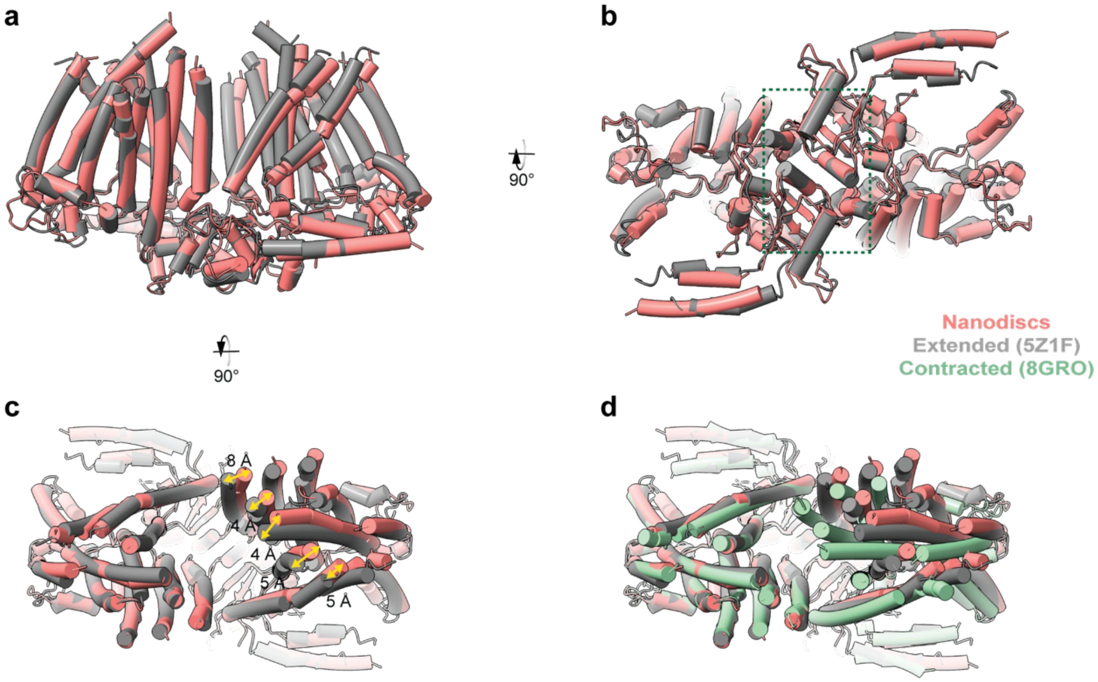

Supplementary Figure 4: Comparison of OSCA3.1 in nanodiscs and detergent states.

a, Front view of model superposition of OSCA3.1 in nanodiscs and extended state in digitonin (PDB: 5Z1F). Alignment done on left protomer. **b,** Intracellular view. Dimerization domain is enclosed in a green square. **c,** Extracellular view illustrating shifts of the nanodisc sample towards the outer edges, and increasing the distance between the two protomers in the inter-subunit cleft. **d,** Extracellular view of the OSCA3.1 in nanodiscs, extended state in digitonin, and contracted state in LMNG (PDB: 8GRO).

bioRxiv preprint doi: https://doi.org/10.1101/2023.10.03.560740; this version posted October 3, 2023. The copyright holder for this preprint (which was not certified by peer review) is the author/funder, who has granted bioRxiv a license to display the preprint in perpetuity. It is made available under aCC-BY 4.0 International license.

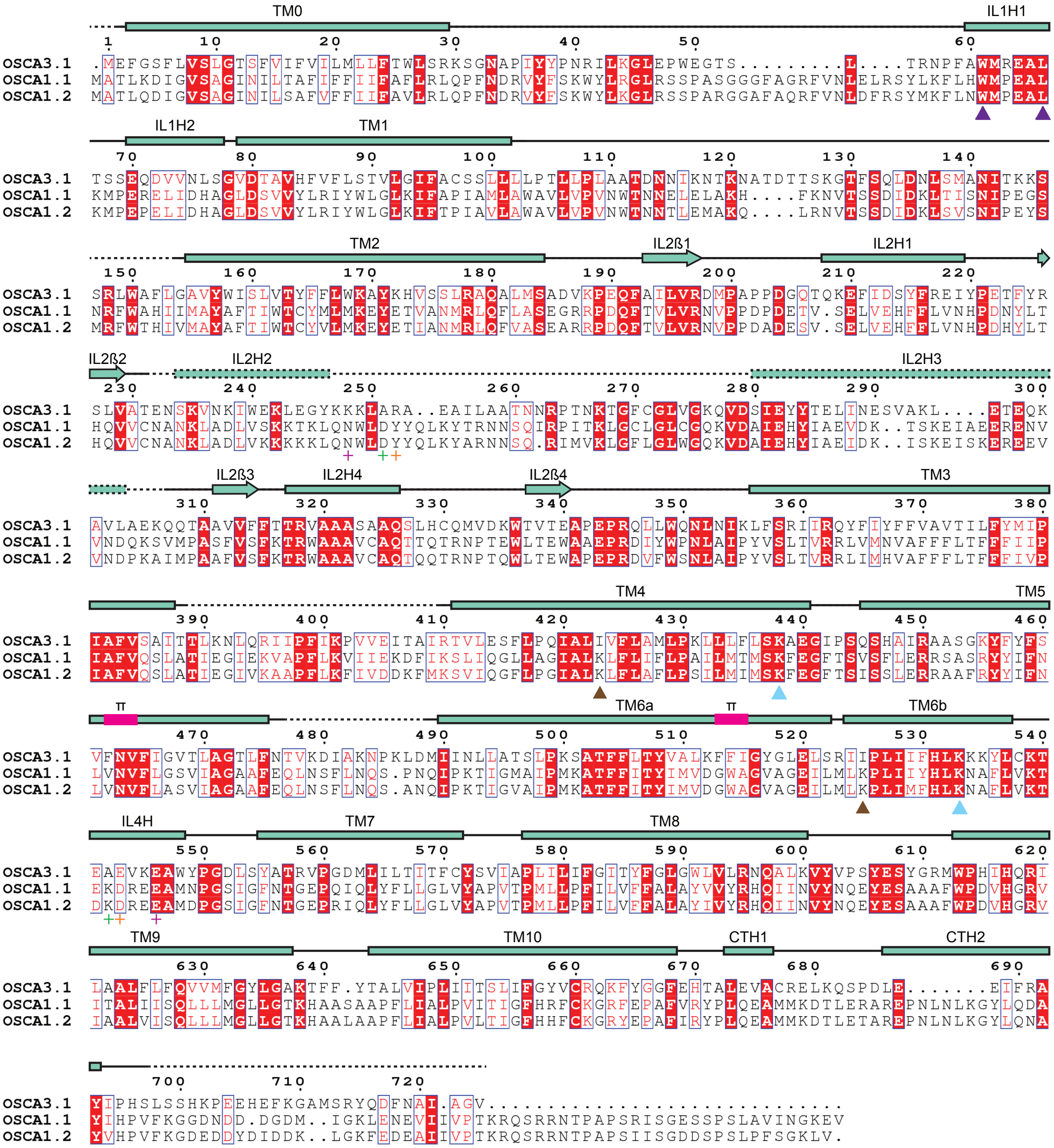

Supplementary Figure 5: Protein sequence alignment of OSCA3.1.
Amino acid sequence alignment of *Arabidopsis thaliana* OSCA3.1, OSCA1.1 and OSCA1.2. Secondary structure of OSCA3.1 represented on top, where rectangles represent α helices and arrows β strands. Dashed lines correspond to regions not modelled. π-helical turns labeled in magenta. IL2H2 and IL2H3 were modelled as poly-Ala and the registry was assigned tentatively. Purple arrowheads point to residues in the amphipathic helix that were mutated in OSCA1.2 for electrophysiology experiments. Predicted lipid-interacting residues denoted by light blue (conserved between OSCA1.2 and OSCA3.1) or brown (not conserved) arrowheads. Evolutionarily coupled residues

bioRxiv preprint doi: https://doi.org/10.1101/2023.10.03.560740; this version posted October 3, 2023. The copyright holder for this preprint (which was not certified by peer review) is the author/funder, who has granted bioRxiv a license to display the preprint in perpetuity. It is made available under aCC-BY 4.0 International license.

marked by plus sign (+), with each pair having the same color. TM: Transmembrane; IL: Intracellular Loop; CT: C-terminal; H: Helix; ß: ß-strand.

bioRxiv preprint doi: https://doi.org/10.1101/2023.10.03.560740; this version posted October 3, 2023. The copyright holder for this preprint (which was not certified by peer review) is the author/funder, who has granted bioRxiv a license to display the preprint in perpetuity. It is made available under aCC-BY 4.0 International license.

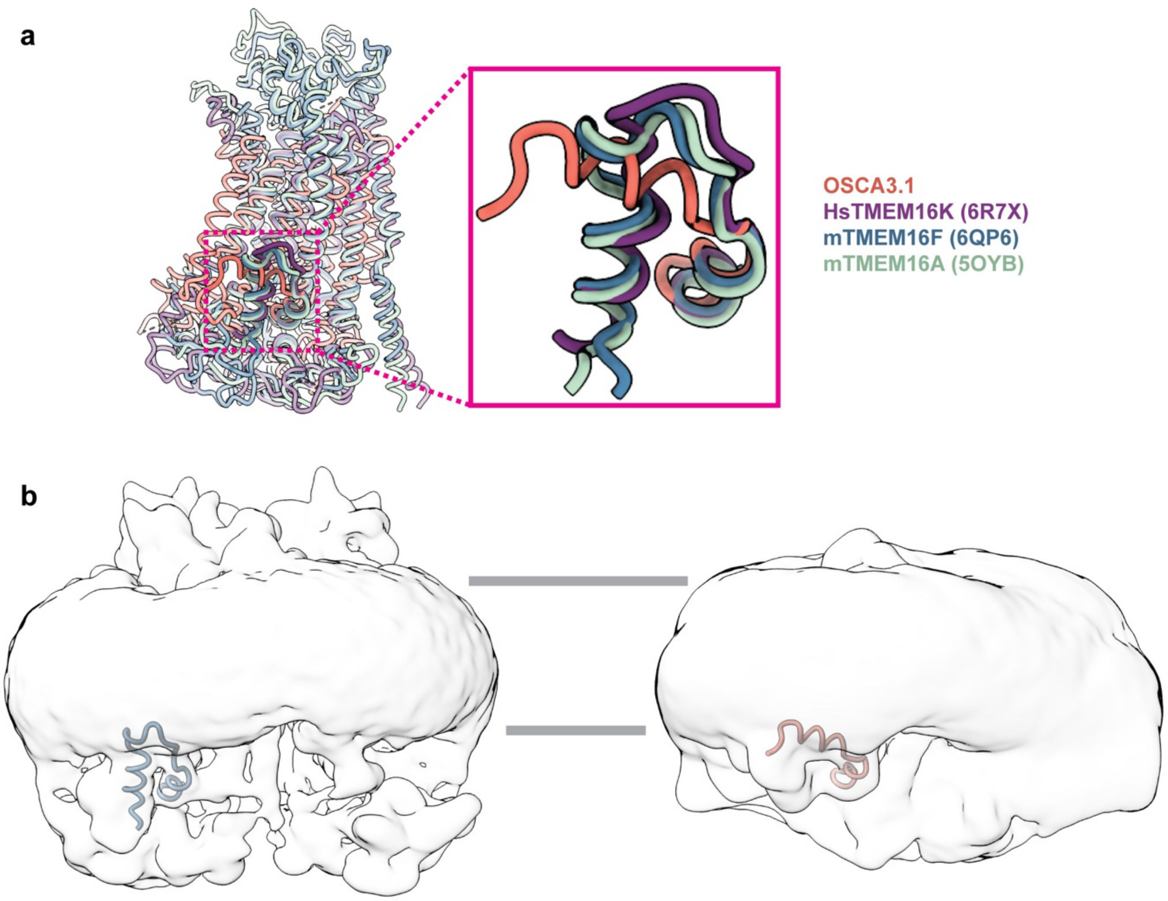

Supplementary Figure 6: Comparison of amphipathic helix of OSCA3.1 with corresponding helices in TMEM16 structures.
a, Superposition of one protomer of OSCA3.1 and selected members of the homologous TMEM16 family. Inset: zoom-in view of amphipathic helix of OSCA3.1 and homologous intracellular helix of TMEM16 homologs. Hs: *Homo sapiens*; m: mouse. **b,** EM density of mTMEM16F (left, EMDB: 4611) and unsharpened density of OSCA3.1 (right) showing position of respective helices shown in **a**. A gaussian-filter of 2 σ was applied to the densities. Horizontal gray lines denote the approximate position of the membrane bilayer.

bioRxiv preprint doi: https://doi.org/10.1101/2023.10.03.560740; this version posted October 3, 2023. The copyright holder for this preprint (which was not certified by peer review) is the author/funder, who has granted bioRxiv a license to display the preprint in perpetuity. It is made available under aCC-BY 4.0 International license.

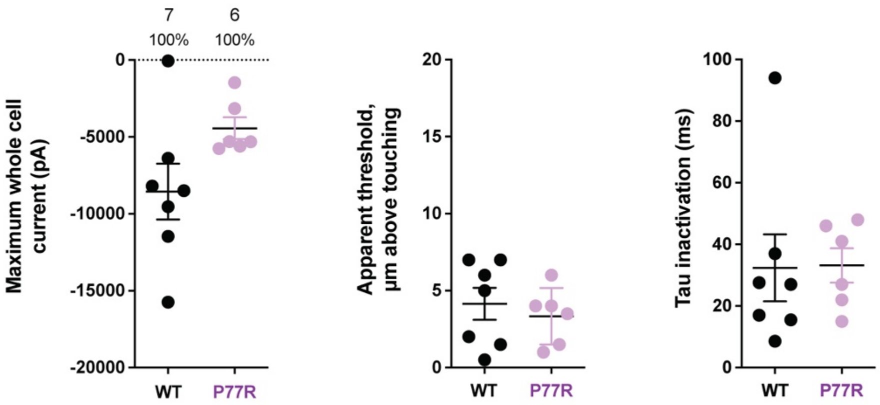

Supplementary Figure 7: Poke-induced response of OSCA1.2$_{P77R}$.
Introduction of arginine from OSCA3.1 into the OSCA1.2 background did not alter the poke-induced response. Left panel: Maximum poke-induced whole-cell currents observed for cells exposed to displacements up to 7.2 ± 1.4 µm (N=7; mean ± S.E.M.) and 6.5 ± 0.6 µm (N=6) above touching for WT and OSCA1.2$_{P77R}$, respectively. Center panel: the apparent threshold in µm above touching for the cells in this cohort. Right panel: Time constant of inactivation of currents elicited in the cells in this cohort.

bioRxiv preprint doi: https://doi.org/10.1101/2023.10.03.560740; this version posted October 3, 2023. The copyright holder for this preprint (which was not certified by peer review) is the author/funder, who has granted bioRxiv a license to display the preprint in perpetuity. It is made available under aCC-BY 4.0 International license.

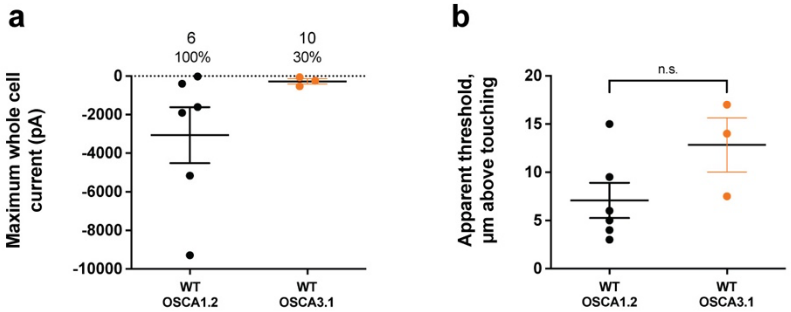

Supplementary Figure 8: OSCA3.1 may be poke-sensitive at high thresholds. Poke-induced whole-cell current responses are observed in HEK-P1KO cells expressing OSCA3.1. **a,** Maximum poke-induced currents observed in whole-cell mode for cells exposed to displacements up to 10.4 ± 1.2 µm (N=6; mean ± S.E.M.) and 13.8 ± 0.9 µm (N=3) above touching for $OSCA1.2_{WT}$ and $OSCA3.1_{WT}$, respectively. Although 6 of 6 OSCA1.2 expressing cells responded to poking, only 3 of 10 showed a response before the disruption of the recording. **b,** The apparent threshold in µm above touching the cell for this cohort.

bioRxiv preprint doi: https://doi.org/10.1101/2023.10.03.560740; this version posted October 3, 2023. The copyright holder for this preprint (which was not certified by peer review) is the author/funder, who has granted bioRxiv a license to display the preprint in perpetuity. It is made available under aCC-BY 4.0 International license.

Supplementary Table 1. Data collection, processing, model refinement and validation.

	OSCA3.1
**Data collection and processing**	
Magnification	29000
Voltage (kV)	300
Electron exposure (e–/Å^2)	50
Defocus range (μm)	-0.4 to -1.5
Pixel size (Å)	1.03
Initial particle images (no.)	1,913,316
Symmetry imposed	C2
Final particle images (no.)	197,944
Map resolution (Å)	2.6
FSC threshold	0.143
Map sharpening *B* factor (Å^2)	-64
**Model**	
Composition	
Peptide chains	2
Protein residues	1148
Ligands	24
R.m.s. deviations	
Bond lengths (Å)	0.023
Bond angles (°)	1.677
Validation	
MolProbity score	0.74
Clashscore	0.74
EMRinger score	4.12
Poor rotamers (%)	0.00
Ramachandran plot	
Favored (%)	98.93
Allowed (%)	1.07
Disallowed (%)	0.00
**Deposition ID**	
EMDB	41911
PDB	8U53
